# Distal Pancreatectomy with and without Celiac Axis Resection for Adenocarcinoma: A Comparison in the Era of Neoadjuvant Therapy

**DOI:** 10.3390/cancers16203467

**Published:** 2024-10-12

**Authors:** Sara K. Daniel, Camille E. Hironaka, M. Usman Ahmad, Daniel Delitto, Monica M. Dua, Byrne Lee, Jeffrey A. Norton, Brendan C. Visser, George A. Poultsides

**Affiliations:** Department of Surgery, Stanford University, Stanford, CA 94305, USA

**Keywords:** modified Appleby procedure, locally advanced pancreas cancer, arterial resection

## Abstract

**Simple Summary:**

Distal pancreatectomy with celiac axis resection (DP-CAR) has equivalent morbidity and survival outcomes compared to standard distal pancreatectomy (DP) in patients with pancreatic adenocarcinoma requiring modern neoadjuvant chemotherapy.

**Abstract:**

Background: Distal pancreatectomy with celiac axis resection (DP-CAR) has been used for selected patients with pancreatic cancer infiltrating the celiac axis. We compared the short- and long-term outcomes between DP-CAR and distal pancreatectomy alone (DP) in patients receiving neoadjuvant therapy. Methods: Patients undergoing DP-CAR from 2013 to 2022 were retrospectively reviewed. Clinicopathologic features, post-operative morbidity, and survival outcomes were compared with patients undergoing DP after neoadjuvant chemotherapy. Results: Twenty-two DP-CAR and thirty-four DP patients who underwent neoadjuvant chemotherapy were identified. There were no differences in comorbidities or CA19-9 levels. OR time was longer for DP-CAR (304 vs. 240 min, *p* = 0.007), but there was no difference in the transfusion rate (22.7% vs. 14.7%). Vascular reconstruction was more common in DP-CAR (18.2% vs. 0% arterial, *p* = 0.05; 40.9% vs. 12.5% venous, *p* = 0.04). There was no difference in morbidity or mortality between the two groups. Although there was a trend towards larger tumors in DP-CAR (5.1 cm vs. 3.8 cm, *p* = 0.057), the overall survival from the initiation of treatment (32 vs. 28 months, *p* = 0.43) and surgery (30 vs. 24 months, *p* = 0.43) were similar. Discussion: DP-CAR is associated with similar survival and morbidity compared to DP patients requiring neoadjuvant chemotherapy and should be pursued in appropriately selected patients.

## 1. Introduction

Despite advances in systemic therapy for pancreatic cancer, the overall 5-year survival for all stages combined is only 12% [1]. For those who undergo surgical resection and adjuvant chemotherapy, the survival rate can increase to over 40%, but a minority of patients are diagnosed with upfront resectable disease [2,3]. In order to increase the number of patients who are surgical candidates, there has been a push to pursue more technically challenging operations that add vascular resections or reconstructions to pancreatectomy. Arterial resections were associated with significantly increased morbidity and mortality in the past; however, advances in surgical technique and in modern chemotherapy for pancreatic adenocarcinoma (PDAC) have helped alter the risk–benefit ratio in favor of more aggressive approaches [4,5,6].

Distal pancreatectomy with celiac axis resection (DP-CAR), also known as the modified Appleby procedure, was first used in 1976 as a treatment for locally advanced pancreatic cancer [7]. The original Appleby procedure was initially described in 1953 as a treatment for gastric cancer with the invasion of the celiac axis [8]. The procedure takes advantage of the retrograde perfusion to the proper hepatic artery via the gastroduodenal artery/pancreatoduodenal arcade from the superior mesenteric artery (SMA), but carries a risk of gastric and hepatic ischemia [7]. Strict criteria exist for who may undergo this procedure for anatomic reasons, but as surgical resection remains the only possibility for cure for PDAC, the number of patients who can benefit from DP-CAR should be maximized [9,10]. Although several studies have described satisfactory long-term outcomes of DP-CAR patients, and many have compared the results to standard distal pancreatectomy (DP) patients without CAR, the rates of neoadjuvant therapy in the comparison DP group have been low [11,12,13,14,15,16,17,18,19,20,21,22,23,24,25,26,27,28,29]. Only one study has reported neoadjuvant therapy administration in more than 50% of the cohort [30].

We evaluated our institutional series of patients who underwent DP-CAR in comparison to those who underwent standard DP after neoadjuvant chemotherapy to see if the addition of celiac artery resection led to acceptable incremental morbidity and an equivalent long-term outcome. DP patients were only included if they had received neoadjuvant chemotherapy to create a comparable control group of patients with a higher risk for PDAC of the body and tail. We evaluated short-term, post-operative outcomes and long-term, overall, and recurrence-free survival.

## 2. Materials and Methods

### 2.1. Study Population

Retrospective clinical chart review was performed following IRB approval for data collection and storage. All patients who had undergone distal pancreatectomy at our institution for PDAC from April 2013 to November 2022 were evaluated. Patients were included if they had undergone DP-CAR or if they had undergone DP only after receiving neoadjuvant chemotherapy. No matching of patients was performed.

### 2.2. Patient Selection and Technical Details

For inclusion, patients needed to be a candidate for a DP anatomically with a tumor in the body or tail of the pancreas. Patients with involvement of the celiac axis (CA) or common hepatic artery (CHA), but no involvement of the GDA or SMA, would be considered for DP-CAR only. Pre-operative CHA embolization was performed selectively, especially if the GDA was felt to be of small caliber and there was no replaced or accessory right hepatic artery noted on pre-operative imaging, to augment pre-operative collateral flow to the liver and stomach. The technique of DP-CAR has been described extensively in previous publications [7,22,31,32]. Additionally, the necessity of additional arterial reconstruction was made intra-operatively at the surgeon’s discretion based on evaluation of gastric and liver perfusion at the completion of the resection.

Patients were selected to undergo neoadjuvant therapy prior to DP if they met borderline resectable criteria by NCCN/AHPBA guidelines or had high-risk features, such as elevated CA19-9 and/or large tumor size, at the discretion of the attending surgeon. Our institutional criteria for determining the length of neoadjuvant therapy are to proceed with pancreatectomy after 3–4 months of neoadjuvant chemotherapy (median of 3 months in this study), provided that adequate biochemical and radiographic responses are demonstrated to allow for safe and margin-negative resection. In cases of poor tolerance to neoadjuvant chemotherapy, surgery was performed sooner, or chemotherapy was switched. We have not adopted the practice of total neoadjuvant therapy (6 months) prior to resection of pancreatic cancer, which has been proposed by some authors but is not universally accepted. The interval from the last dose of chemo to surgery is typically 4 weeks at our center. Our institutional practice is to assess peritoneal cytology selectively, in the presence of very high serum CA19-9 (>1000 U/mL) or suspicious peritoneal nodularity on cross-sectional imaging, typically before initiation of neoadjuvant therapy. If a patient was noted to have positive peritoneal cytology, resection was not pursued even after great response to chemotherapy.

### 2.3. Outcomes

For patients who underwent neoadjuvant therapy, the change in CA 19-9 levels was calculated from the value prior to treatment and the last value prior to surgery. Post-operative complications were graded according to the Clavien–Dindo (CD) classification, with major complications defined as grade CD IIIa or above. Post-operative pancreatic fistula (POPF) was defined according to the classification of the International Study Group for Pancreas Surgery (ISGPS), with clinically significant POPF being ISGPS grade B or C. Post-operative hepatic ischemia was defined as 5-fold post-operative elevation of transaminase levels with any associated radiographic ischemic infarction or with associated hepatic insufficiency. Post-operative gastric ischemia was defined as post-operative gastric dysfunction with endoscopic evidence of ischemic gastropathy, partial or transmural necrosis, perforation, or need for reoperation to address gastric necrosis. R0 resection status was defined as no tumor cells within 1 mm of the final margin. Overall and recurrence-free survival were calculated from the date of surgery and from the date of initiation of neoadjuvant treatment.

### 2.4. Statistics

For continuous variables, median value with IQR was reported and assumptions were tested using Mann–Whitney U tests. For categorical data, percentages of groups were reported, and assumptions tested using chi-square tests, or Fischer’s exact test if the chi-square assumptions were not met. For survival analysis, Kaplan–Meier curves and log-rank tests were used. Univariate regression analyses were performed using SPSS generalized linear modeling (IBM, Chicago, IL, USA). Statistical analysis was performed using Stata 18 (StataCorp, College Station, TX, USA) and Jamovi 2.6.2 (GNU general public license). A value of *p* < 0.05 was considered significant.

## 3. Results

From 2013 to 2022, a total of 102 patients with body/tail PDAC were examined. Twenty-two patients underwent DP-CAR, two of whom did not receive neoadjuvant chemotherapy. Of the remaining 80 patients who underwent DP, 34 patients received neoadjuvant chemotherapy. This constituted our control group as we wanted to a comparable cohort of “higher risk” DP patients for whom the treating team elected to proceed with neoadjuvant chemotherapy. The DP-CAR and DP post-neoadjuvant groups were very similar in pre-operative characteristics (Table 1), including demographics and comorbidities. Pre-operative lab values were not different between the two groups. There was no significant difference between tumor size either before initiation or after completion of neoadjuvant chemotherapy. Similarly, CA 19-9 levels were comparable in the two groups both at diagnosis (396 vs. 224 U/mL, *p* = 0.72) and after neoadjuvant therapy (41 vs. 45 U/mL, *p* = 0.42), indicating a similar tumor burden and response to treatment between the two groups.

Regarding neoadjuvant treatment, 91% of the DP-CAR and 100% of the DP patients in our cohort received neoadjuvant chemotherapy. The duration of neoadjuvant chemotherapy was similar in the two groups (3.0 months vs. 3.0 months, *p* = 0.86). The most common regimen in both groups was FOLFIRINOX (59.1% vs. 76.4%), followed by gemcitabine and abraxane (27.2% and 32.3%). One patient in the DP group received neoadjuvant immunotherapy after pre-operative biopsy demonstrated high PD-L1 expression and was subsequently found to be deficient in MSH2 and MSH6. There was no significant difference in the utilization of neoadjuvant radiation, with 13.6% in the DP-CAR and 14.7% in the DP group (*p* = 1.0). All patients who received neoadjuvant radiation received it in addition to neoadjuvant systemic chemotherapy.

To increase the collateral supply through the pancreaticoduodenal arcade, pre-operative embolization of either hepatic or left gastric artery was performed 1–2 weeks before surgery in seven (31.8%) DP-CAR patients. One DP patient received pre-operative splenic artery embolization to pre-operatively decompress gastric varices. Interestingly, nine (40.9%) DP-CAR patients had a replaced or accessory right hepatic artery noted on pre-operative imaging. Splenic artery involvement, while seen radiographically in 100% of the DP-CAR group, was only seen in 62% of the DP group.

A comparison of intra-operative characteristics is noted in Table 2. The amount of IV fluids received intra-operatively (3.5 L vs. 2.3 L, *p* = 0.002) and the procedure duration were higher in the DP-CAR group (304 vs. 240 min, *p* = 0.007). The difference in estimated blood loss (EBL), however, did not reach significance with a median of 575 and 400 mL, respectively (*p* = 0.18), and there was no difference in the percentage of patients who received intra-operative blood transfusion (*p* = 0.49). Diagnostic laparoscopy was performed in 31.8% in the DP-CAR group and 20.5% in the DP group (*p* = 0.36). One DP case was performed laparoscopically. Both arterial (18.2% vs. 0%, *p* = 0.02) and venous (40.9% vs. 11.7%, *p* = 0.02) reconstruction rates were higher in the DP-CAR group. Of the four DP-CAR patients who had proper hepatic arterial reconstruction, two were repaired with primary end-to-end anastomosis and two required a bypass graft using greater saphenous vein. Venous reconstruction was most commonly performed at the portal vein (PV)—superior mesenteric vein (SMV) confluence. An interposition graft was performed in two (9.1%) DP-CAR patients using a cryopreserved vein for one patient and an internal jugular vein for the other. Additionally, two (2.9%) DP patients underwent an interposition graft using an internal jugular vein for one patient and the left renal vein for the other. End-to-end vein repair was performed in 13.6% of DP-CAR patients and 0.0% of DP patients. Patch angioplasty using bovine pericardium or inferior mesenteric vein (IMV) was performed in 18.2% of DP-CAR patients and 5.9% of DP patients. Concomitant organ resection was required in 54.5% of DP-CAR patients and 50.0% of DP patients (*p* = 0.67), with left adrenalectomy being the most common in DP-CAR (27.2% vs. 14.7%). Partial gastrectomy was performed in 22.7% of DP-CAR and 23.5% of DP patients.

Regarding pathologic data (Table 3), there was a trend towards larger tumor size in the DP-CAR group (5.1 cm vs. 3.8 cm, *p* = 0.057). Tumor differentiation (*p* = 0.16), lymphovascular (*p* = 1.0) and perineural (*p* = 0.29) invasion rates, and the rate of lymph node metastasis (*p* = 0.56) were not different between the two groups. Importantly, the R0 resection rate was similar between the two groups (91% after DP-CAR and 100% after DP, *p* = 0.16). There was no significant difference between groups for tumor response to neoadjuvant therapy.

Post-operative characteristics are compared in Table 4. There was no 30-day post-operative mortality in either group, and only one death within 90 days in the DP group. Major morbidity, defined as Clavien–Dindo complication grade IIIa or higher, was not different between the two groups (36.3% vs. 26.4%, *p* = 0.55). Both hepatic (13.6% vs. 0%, *p* = 0.056) and gastric ischemia (13.6% vs. 2.9%, *p* = 0.29) were more common in the DP-CAR group but these differences did not reach statistical significance. Peak post-op transaminase levels were statistically higher after DP-CAR than DP, but the median levels remained clinically not concerning (AST 182 vs. 86, *p* = 0.009; ALT 122 vs. 56, *p* = 0.007). The post-operative ICU admission rate after DP-CAR was 31.8% compared to 11.7% (*p* = 0.089) after DP. Both groups had one patient requiring unplanned return to the OR (*p* = 1.0). There were no ISGPS grade C fistulas, and no significant difference in rate of grade B fistulas between the two groups (31.8% vs. 14.7%, *p* = 0.08). Two DP-CAR patients developed anastomotic leaks requiring IR drainage, one after a partial gastrectomy and one after a colonic resection. The median length of stay (LOS) was longer for the DP-CAR group (7.5 vs. 6.0 days, *p* = 0.029).

There was no significant difference in the percentage of patients who received adjuvant systemic chemotherapy (68% vs. 79%, *p* = 0.33), radiation (27% vs. 15%, *p* = 0.31), or immunotherapy (5% vs. 9%, *p* = 1.0).

**Figure 1:**
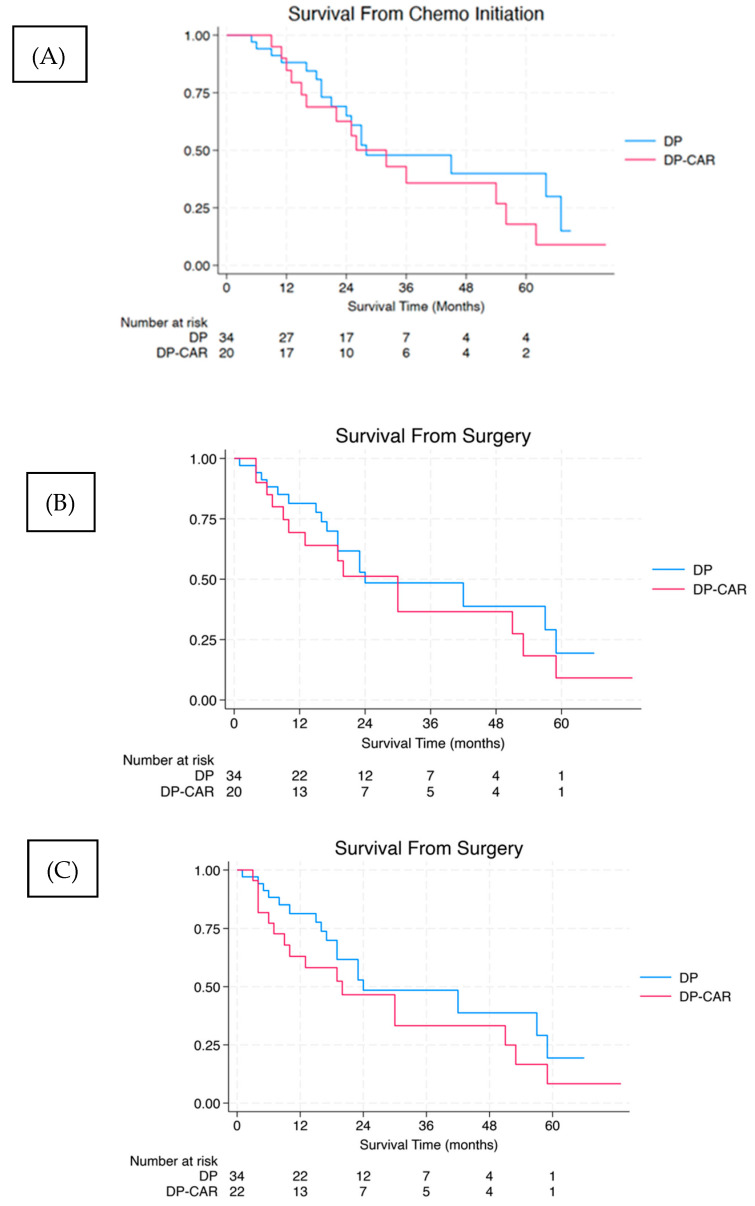
Overall survival in patients after DP and DP = CAR. Kaplan–Meier survival curves for overall survival from (**A**) time of initial treatment and (**B**) surgery excluding the two DP-CAR patients who did not receive neoadjuvant chemotherapy. (**C**) Overall survival from time of surgery excluding the two DP-CAR patients who did not receive neoadjuvant chemotherapy. DP = distal pancreatectomy, DP-CAR = distal pancreatectomy with celiac axis resection.

Median follow-up time post-operatively was 16.5 months for the DP-CAR and 18 months for the DP groups. For survivors, the median follow-up time was 19.5 and 20 months post-operatively for the DP-CAR and DP groups, respectively. Excluding the two patients in the DP-CAR group who did not receive neoadjuvant chemotherapy, the median overall survival (OS) was 30 months from surgery and 32 months from the initiation of therapy compared to 24 months from surgery and 28 months from the initiation of treatment after DP; neither of these differences reached significance (*p* = 0.43 for both Figure 1). When the two patients who did not receive neoadjuvant chemotherapy are included in the DP-CAR group, the median survival after surgery decreases to 26 months (*p* = 0.24).

## 4. Discussion

In this study, we have demonstrated that DP-CAR for pancreatic adenocarcinoma can provide a similar long-term survival benefit to standard DP for patients requiring neoadjuvant therapy without significantly increased morbidity or mortality. To compare our results with previously published studies, we performed a review of publications where at least 20 patients undergoing DP-CAR were described in the last 10 years (Table 5) [11,12,13,14,15,16,17,18,19,20,21,22,23,24,25,26,27,28,29]. Our cohort seemed to be equivalent in case complexity regarding the need for additional venous resection (41% in our study, range 14–73% in relevant studies), arterial reconstruction (18%, range 0–83%), and concomitant visceral resection (55%, range 10–80%). Both tumor size (5.1 cm, range 3.0–5.3 cm) and R0 resection rate (91%, range 50–93%) in our study were towards the higher end of ranges for the comparison studies. We had no 90-day mortality in our DP-CAR patients, whereas this ranged from 0 to 16% in other studies. Major morbidity was 36% in our study compared to 13–60% in others. Our median LOS of 7.5 days after DP-CAR was favorable compared to other reports (8–33.5 days). Our median OS for patients undergoing DP-CAR was 32 months from treatment initiation and was consistent with prior studies (range 16–35 months).

Hepatic artery embolization was performed pre-operatively in seven of our twenty-two patients undergoing DP-CAR; two of these patients still suffered hepatic ischemia post-operatively and one had additional gastric ischemia, all of which were managed conservatively. Truty et al. similarly found a significant increase in both hepatic and gastric ischemia in patients that had undergone pre-operative embolization; however, in their study, 32% of patients underwent total pancreatectomy and 62% underwent arterial revascularization [19]. Conversely, Storkholm et al. did not see ischemic complications with high rates of embolization, but also routinely used epoprostenol, a vasodilator used to treat pulmonary arterial hypertension in the post-operative setting, which may have decreased ischemia rates [18]. Shin et al. did not perform any pre-operative embolization in their study of 48 patients; however, one patient required reoperation for gallbladder infarction and one patient required gastrectomy for extensive devascularization [29]. Ueda et al. specifically addressed this question by evaluating their institutional dataset of 31 patients in 2019. They did not find a correlation with pre-operative embolization or the time between embolization and surgery and liver ischemia after DP-CAR [32]. Overall, our findings and data from the aforementioned studies agree that pre-operative CHA embolization may not be entirely protective against hepatic or gastric ischemia. However, our institutional preference has been to perform pre-operative CHA if the GDA is felt to be of small caliber and there is no replaced or accessory right hepatic artery noted on pre-operative imaging. Alternatively, interventional radiology can measure the blood flow in the proper hepatic artery after temporarily occluding the celiac artery to assess the need for pre-op CHA embolization.

Many of the previous studies evaluating DP-CAR suggested that neoadjuvant chemotherapy was associated with an increased R0 resection rate [14,17,19,29]. In turn, R0 resection rate was associated with increased survival in many studies; four of the five studies with the lowest R0 resection rates also had a median OS less than 20 months [13,14,15,20]. In some studies, such as in Truty et al., neoadjuvant chemotherapy was associated with increased RFS and OS, whereas in others, such as in Inoue et al., only OS was increased with neoadjuvant chemotherapy [16,17,19,22]. In addition to improved survival, Yoshiya et al. found lower rates of arterial invasion and fewer positive lymph nodes after neoadjuvant chemotherapy [17]. Similarly, neoadjuvant chemotherapy was associated with decreased lymphovascular invasion and N0 rates in the study by Shin et al. [29]. There was no increased morbidity or mortality associated with receiving neoadjuvant chemotherapy in any study [17,19]. These data support the routine use for contemporary neoadjuvant chemotherapy for patients under consideration for DP-CAR.

Five of the studies on DP-CAR outcomes had a comparison DP group. As expected, the EBL, OR time, and tumor size were increased in DP-CAR patients compared to DP in most studies, as well as morbidity and LOS [13,18,23,25,27]. More DGE was generally seen in the DP-CAR group [25,27]. Additionally, Liu et al. found that perineural invasion and positive LNs were higher in DP-CAR compared to DP [27]. Overall, fewer DP patients received neoadjuvant chemotherapy than DP-CAR, although this difference did not reach significance [13,23,25]. Survival outcome differences between DP and DP-CAR were mixed between the five studies. Two studies had worse OS for DP-CAR than DP [13,23], and three studies showed no difference in the median OS between DP and DP-CAR [18,25,27]. Similarly, our study, which used a control group of DP patients requiring neoadjuvant chemotherapy, showed equivalent survival outcomes between DP and DP-CAR, reinforcing the role of this procedure in carefully selected patients, following disease control with neoadjuvant chemotherapy.

The limitations of this study include its retrospective nature and limited follow-up for some patients; this is not unexpected due to referral patterns to our institution, which provides quaternary complex oncologic surgical care for a large geographic area. As such, patients return to their local medical oncologists for surveillance after the completion of adjuvant therapy. This also limits the ability for the evaluation of longer-term quality of life metrics such as diarrhea. The smaller number of patients in the control group perhaps limited the statistical power; however, we felt that limiting the DP group to patients who had received neoadjuvant chemotherapy helped create a more comparable group for comparison to the DP-CAR patients. Although the short-term and long-term outcomes between the two groups were similar, there was undoubtedly selection bias involved in the pre-operative decision making especially for the DP-CAR patients given the critical nature of the operation. There were also a significantly higher percentage of patients in the DP-CAR group with aberrant right hepatic artery anatomy compared to the DP group, and this may have played a role in our favorable outcomes after DP-CAR.

## 5. Conclusions

In summary, our single-institution experience with DP-CAR patients demonstrates no difference in perioperative morbidity, mortality, and long-term survival after DP-CAR compared to DP alone for patients undergoing neoadjuvant chemotherapy. As this operation is being increasingly performed for patients with traditionally “unresectable” pancreatic cancer encasing the celiac axis, clinicians should be aware of its equivalent short- and long-term outcomes when compared with similar cases without celiac axis encasement. As long-term quality of life data also become available, indicating intact nutritional status and a lack of intractable diarrhea within the first-year post-surgery, DP-CAR remains a reliable and valuable component of the pancreatic surgeon’s armamentarium against locally advanced pancreatic cancer [33].

## Figures and Tables

**Table 1:** Pre-operative characteristics.

	DP-CAR	DP	
# Patients	22	34	
Weight (kg)	69.1 (52–113)	75.2 (47–104)	*p* = 0.397
BMI (kg/m^2^)	23.7 (19–35)	25.4 (19–35)	*p* = 0.763
Age at Surgery (years)	66.5 (42–82)	68.0 (44–85)	*p* = 0.687
Male	13 (59.1%)	21 (61.7%)	*p* = 1.00
Race			*p* = 0.759
Caucasian/White	15 (68.2%)	22 (64.7%)	
AAPI	3 (13.6%)	8 (23.5%)	
Black	0 (0.0%)	0 (0.0%)	
Hispanic/Latino	3 (13.6%)	3 (8.8%)	
Other	1 (4.5%)	1 (2.9%)	
Charlson Comorbidity Index	4.5 (3–8)	5.0 (2–7)	*p* = 0.842
ASA Class			*p* = 0.516
2	4 (18.2%)	10 (29.4%)	
3	18 (81.8%)	23 (67.6%)	
4	0 (0.0%)	1 (2.9%)	
Pre-operative Weight Loss > 10%	11 (50.0%)	10 (29.4%)	*p* = 0.161
Current Smoker	2 (9.1%)	5 (14.7%)	*p* = 1.00
Neoadjuvant Chemotherapy	20 (90.9%)	34 (100%)	*p* = 0.150
Months	3.0 (2–7)	3.0 (1.5–10)	*p* = 0.869
FOLFIRINOX or FOLFOXIRI	13 (59.1%)	26 (76.4%)	
Gemcitabine/Abraxane	5 (22.7%)	11 (32.3%)	
Other	2 (9.1%)	3 (8.8%)	
Neoadjuvant Radiation	3 (13.6%)	5 (14.7%)	*p* = 1.00
Neoadjuvant Immunotherapy	0 (0.0%)	1 (2.9%)	*p* = 1.00
Surgery Specific Factors			
Prior Pancreatic Surgery	1 (4.5%)	1 (2.9%)	*p* = 1.00
**Radiographic Splenic Artery Involvement**	**22 (100%)**	**21 (61.8%)**	***p*** ** = 0.001**
**Aberrant Right Hepatic Artery**	**9 (40.9%)**	**4 (11.7%)**	***p*** ** = 0.021**
**Any Pre-operative Embolization**	**7 (31.8%)**	**1 (2.9%)**	***p*** ** = 0.004**
Diagnosis radiographic tumor size (cm)	4.7 (2.4–11.0)	4.0 (2.0–7.0)	*p* = 0.236
Pre-op radiographic tumor size (cm)	3.4 (0.6–5.5)	3.2 (1.3–5.6)	*p* = 0.506
Labs			
Serum Creatinine (mg/dL)	0.80 (0.5–1.0)	0.71 (0.3–1.5)	*p* = 0.632
Albumin (g/dL)	3.80 (2.1–4.8)	4.0 (2.3–4.8)	*p* = 0.219
Total Bilirubin (mg/dL)	0.30 (0.2–1.7)	0.40 (0.2–0.9)	*p* = 0.828
AST (U/L)	30 (14–79)	27 (14–77)	*p* = 0.447
ALT (U/L)	37 (20–112)	32 (16–150)	*p* = 0.118
Alkaline Phosphatase (IU/L)	98 (53–221)	113 (41–886)	*p* = 0.478
INR	1.0 (1.0–1.6)	1.1 (0.9–1.3)	*p* = 0.972
Hematocrit	35.9 (27–46)	36.7 (25–44)	*p* = 0.580
Platelets (10^9^/L)	182 (69–390)	165 (60–520)	*p* = 0.621
Tumor Markers			
Diagnosis CA 19-9	396 (2–3942)	224 (1–62,000)	*p* = 0.727
Pre-op CA 19-9	41 (4–1067)	45 (1–3361)	*p* = 0.424
% Decrease in CA 19-9	76% (−731–100%)	77% (−181–100%)	*p* = 0.631

The bold denotes significant values.

**Table 2:** Intra-operative characteristics.

	DP-CAR	DP	
# Patients	22	34	
Epidural	14 (63.6%)	13 (38.2%)	*p* = 0.100
**Procedure Time (minutes)**	**304 (209–626)**	**240 (129–520)**	***p*** ** = 0.007**
EBL (mL)	575 (50–11,000)	400 (20–4700)	*p* = 0.069
Fluids in OR			
**IVF (mL)**	**3500 (1200–15,500)**	**2250 (600–7250)**	***p*** ** = 0.002**
RBC Transfusion (% of patients)	5 (22.7%)	5 (14.7%)	*p* = 0.491
Concomitant Organ Resection	12 (54.5%)	17 (50.0%)	*p* = 0.789
Left Adrenalectomy	6 (27.2%)	5 (14.7%)	
Partial Gastrectomy	5 (22.7%)	8 (23.5%)	
Partial Colectomy	2 (9.1%)	2 (5.9%)	
Cholecystectomy	3 (13.6%)	8 (23.5%)	
Small Bowel Resection	1 (4.5%)	2 (5.9%)	
Liver Wedge Resection	1 (4.5%)	0 (0.0%)	
Diagnostic Laparoscopy	7 (31.8%)	7 (20.5%)	*p* = 0.363
**Arterial Reconstruction**	**4 (18.2%)**	**0 (0.0%)**	***p*** ** = 0.020**
Bypass	2 (9.1%)		
End-to-end repair	2 (9.1%)		
**Venous Reconstruction**	**9 (40.9%)**	**4 (11.7%)**	***p*** ** = 0.021**
Bypass	2 (9.1%)	2 (5.9%)	
End-to-end Repair	3 (13.6%)	0 (0.0%)	
Patch Repair	4 (18.2%)	2 (5.9%)	

The bold denotes significant values.

**Table 3:** Pathology.

	DP-CAR	DP	
# Patients	22	34	
Tumor Size (cm)	5.1 (1.5–11.5)	3.8 (1.5–8.5)	*p* = 0.057
Differentiation			*p* = 0.163
Well	4 (18.1%)	8 (23.5%)	
Moderate	10 (45.4%)	20 (58.8%)	
Poor	4 (18.1%)	4 (11.7%)	
Unable to Assess	4 (18.1%)	2 (5.9%)	
Invasion			
Lymphovascular	7 (31.8%)	11 (32.3%)	*p* = 1.00
Perineural	17 (77.3%)	30 (88.2%)	*p* = 0.294
Positive LNs	16 (72.7%)	21 (61.7%)	*p* = 0.564
Resection Status			
R0	20 (90.9%)	33 (100%)	*p* = 0.156
R1	2 (9.1%)	0 (0.0%)	
Tumor Response Grade			*p* = 0.886
1 (Well)	1 (9.1%)	4 (13.3%)	
2 (Moderate)	7 (31.8%)	16 (53.3%)	
3 (Poor)	3 (27.3%)	10 (33.3%)	

**Table 4:** Post-operative characteristics.

	DP-CAR	DP	
# Patients	22	34	
**LOS (days)**	**7.5 (5–94)**	**6.0 (3–22)**	***p*** ** = 0.029**
Post-operative ICU	7 (31.8%)	4 (11.7%)	*p* = 0.089
Mortality with 30 days of Surgery	0 (0.0%)	0 (0.0%)	*p* = 1.00
Mortality with 90 days of Surgery	0 (0.0%)	1 (2.9%)	*p* = 1.00
Complications within 30 Days of Surgery			
Superficial Incisional SSI	4 (18.2%)	2 (5.8%)	*p* = 0.198
Deep Incisional SSI	1 (4.5%)	1 (2.9%)	*p* = 1.00
Organ Space SSI	9 (40.9%)	9 (26.4%)	*p* = 0.380
PE	1 (4.5%)	2 (5.9%)	*p* = 1.00
Transfusion	5 (22.7%)	5 (14.7%)	*p* = 0.491
Unplanned Return to OR	1 (4.5%)	1 (2.9%)	*p* = 1.00
Chyle Leak	2 (9.1%)	3 (8.8%)	*p* = 1.00
TPN	5 (22.7%)	5 (15.7%)	*p* = 0.491
Hepatic Ischemia	3 (13.6%)	0 (0.0%)	*p* = 0.056
Gastric Ischemia	3 (13.6%)	1 (2.9%)	*p* = 0.289
**Post-operative Weight Loss > 10%**	**11 (50.0%)**	**7 (20.6%)**	***p*** ** = 0.039**
Morbidity (C-D IIIa or greater)	8 (36.3%)	9 (26.4%)	*p* = 0.554
Readmission Within 30 Days of Discharge	4 (18.2%)	9 (26.4%)	*p* = 0.535
ISGPF Fistula Grade B or C	7 (31.8%)	5 (14.7%)	*p* = 0.089
Recurrence	18 (81.8%)	20 (58.8%)	*p* = 0.087
Adjuvant Chemotherapy	15 (68.1%)	27 (79.4%)	*p* = 0.334
Months	2.5 (0.5–24)	3.5 (0–15)	*p* = 0.499
FOLFIRINOX or FOLFOXIRI	5 (22.7%)	10 (29.4%)	
Gemcitabine/Abraxane	4 (18.2%)	7 (20.6%)	
Gemcitabine	4 (18.2%)	1 (2.9%)	
Other	4 (18.2%)	8 (23.5%)	
Adjuvant Radiation	6 (27.2%)	5 (14.7%)	*p* = 0.310
Adjuvant Immunotherapy	1 (4.5%)	3 (8.8%)	*p* = 1.00
Post-op Labs			
**Peak AST**	**182 (26–4285)**	**86 (21–274)**	***p*** ** = 0.009**
**Peak ALT**	**122 (33–3366)**	**56 (18–362)**	***p*** ** = 0.007**

The bold denotes significant values.

**Table 5:** Previous DP-CAR studies.

First Author	# DP-CAR Patients	Neo-Adjuvant Therapy	Pre-Operative Embolization	Venous Resection	Arterial Resection	Visceral Resection	Tumor Size (cm)	Positive Lymph Nodes	R0 Resection	Major Morbidity	Median LOS (days)	90-Day Mortality	Median Overall Survival (months)
Current Study	22	91%	32%	41%	18%	55%	5.1	73%	91%	36%	7.5	0%	26
Shin	48	60%		31%	4%	19%	3.2	67%	75%	25%	13	2%	25.5
Loos	71	61%	0%	44%	10%	55%		79%	58%	32%	18	%	28
Liu	45	13%		49%	7%	80%	5.0	58%	80%	33%	21	2%	20.8
Ergorov	40	53%	0%	38%	20%	10%	5.2	80%	93%	13%	14	8%	29
Li	21	43%	0%	14%	0%		5.3	81%	71%	38%	16.7	5%	27.4
Schmoker	54	98%			17%	30%	3.0	35%	87%	19%	8	2%	25
Murakami	32	100%	94%	44%	9%	38%	3.1	69%	81%	56%		3%	37
Inoue	55	47%		25%	44%			73%	85%	60%	32		33.1
Ramia	45	69%	37%				4.1	8%	80%	49%	22	16%	13.7
Addeo	60	85%		73%	83%			78%	50%	32%		5%	17.7
Truty	90	93%	4%	66%	62%	80%		22%	88%	53%	12.5	10%	36.2
Storkholm	21	14%	71%	29%			4.1	76%	76%	28%	11.2	0%	23.5
Yoshiya	20	55%	80%	40%			4.2	70%	75%	40%	33.5	0%	30.7
Yoshitomi	31	100%	77%	39%		19%	4.1		74%	42%	32	3%	38.6
Klompmaker	68	50%	22%	27%	13%		4.0	66%	53%	25%	16.5	16%	17
Okada	50	52%	92%	26%			3.0	62%	62%	42%	21	8%	16
Yamamoto	72	56%		33%			3.5	69%	67%	42%		4%	17.5
Nakamura	80	20%		61%	6%	16%		63%	93%	41%	38	5%	30.9
Ocuin	30	96%		47%			4.2	50%	80%	35%	10.7	14%	35

## Data Availability

The data presented in this study are available on request from the corresponding author due to institutional policy.
